# Effects of Transglutaminase and Epigallocatechin Gallate on the Structural and Physicochemical Properties of Fish Skin Gelatin from *Takifugu rubripes*

**DOI:** 10.3390/gels11090725

**Published:** 2025-09-11

**Authors:** Lingyu Han, Yulong Zhang, Bing Hu, Ying Zhang, Jijuan Cao, Jixin Yang, Saphwan Al-Assaf

**Affiliations:** 1 Key Lab of Biotechnology and Bioresources Utilization of Ministry of Education, College of Life Science, Dalian Minzu University, Dalian 116600, China; hanlingyu1001@126.com (L.H.); yulong20916@163.com (Y.Z.); hlbehu@163.com (Y.Z.); 20191414@dlnu.edu.cn (J.C.); 2 Faculty of Social and Life Sciences, Wrexham University, Plas Coch, Mold Road, Wrexham LL11 2AW, UK; j.yang@glyndwr.ac.uk; 3 Hydrocolloids Research Centre, University of Chester, Chester CH1 4BJ, UK

**Keywords:** gelatin, *Takifugu rubripes*, transglutaminase, epigallocatechin gallate, rheology

## Abstract

Fish skin gelatin (FG) has garnered considerable attention as a potential substitute for mammalian gelatin. In this study, *Takifugu rubripes* skin gelatin was chemically modified using transglutaminase (TG) and epigallocatechin gallate (EGCG). Subsequently, the rheological, structural, and physicochemical properties of FG modified with varying concentrations of TG and EGCG were systematically examined and compared. As the concentrations of TG and EGCG increased, more extensive interactions occurred in FG, leading to a significant enhancement of gelatin properties. Following modification, the molecular weight of FG proteins increased, and this was accompanied by enhanced surface hydrophobicity and gel strength. Rheological analysis further demonstrated that the viscosity of FG modified with TG and EGCG was higher than that of unmodified FG and was positively correlated with the treatment concentrations of TG and EGCG. Additionally, the results indicated that the effect of TG modification was more pronounced than that of EGCG modification. Overall, this study demonstrates that both TG and EGCG modifications can effectively overcome the inherent limitations of fish skin gelatin, with TG showing superior efficiency as a cross-linking agent. The enhanced thermal stability, gel strength, and rheological properties achieved through these interactions significantly expand the potential applications of fish gelatin in the food industry, making it a more viable alternative to mammalian gelatin.

## 1. Introduction

Gelatin is a fibrous protein obtained through the partial denaturation and hydrolysis of collagen, which is present in animal skins and bones [1]. Although gelatin has a similar amino acid composition as collagen, it exhibits distinct rheological, emulsification, and gelation properties [2]. Owing to its exceptional gelling and water-holding ability, as well as its emulsifying properties, foaming capacity, and biocompatibility, gelatin has found wide applications in multiple areas, including food, cosmetics, pharmaceuticals, and packaging [3].

Compared with major freshwater fish species such as grass carp, common carp, snakehead, and bighead carp, pufferfish exhibit significantly higher economic value. At present, pufferfish processing remains largely confined to the primary stages of live or ice-fresh sales, generating substantial by-products during processing, including fish bones (32%), fish skin (16%), liver (8%), testis (up to 20%, varying seasonally), and fins (3%) [4]. Over 60% of these by-products are ultimately discarded, while only approximately 30% of the fish is directly edible. These by-products are rich in collagen, polyunsaturated fatty acids, protamine, and various other nutritional and functional components [5], and hold considerable potential for application in the fields of functional foods, medical foods, and pharmaceuticals. Therefore, elucidating the nutritional value and development potential of pufferfish, addressing the current limitations such as limited product diversity, excessive by-product waste, and enhancing the overall added value of pufferfish, has become a critical challenge that needs to be resolved in the pufferfish industry. The crude protein content in pufferfish skin ranges from 20.17% to 37.61%, with *Takifugu rubripes* skin containing up to 35.07% crude protein [6], making it an excellent raw material for gelatin production. The gelatin extraction yield from *Takifugu rubripes* skin ranges between 28.1% and 41.5%, which is comparable to that of pink perch (27.3%) and chum salmon skin (26.1–43.5%), but lower than that of seabass (51.6–66.4%) and Nile perch (64.3%) skin [7]. The extraction yield increases as the extraction temperature rises from 45 °C to 65 °C, and extending the extraction time at a constant temperature further enhances the yield. The isoelectric point of *Takifugu rubripes* skin gelatin is 6.74, which is similar to that of seabass (6.6) and unicorn leatherjacket skin gelatin, yet lower than that of tilapia skin gelatin (8.3) and higher than that of squid gelatin (5.94) [8]. This suggests that *Takifugu rubripes* skin gelatin may contain a slightly higher proportion of acidic amino acids. However, FG has a lower content of proline and hydroxyproline than mammalian gelatin [9], which leads to a reduced gelling capacity, lower melting point, and weaker gel strength [10]. Consequently, higher concentrations of FG are required for effective gel formation.

To address these shortcomings, scientists have modified FG via enzymatic [11], chemical cross-linking [12], and physical treatments [13]. Moreover, polysaccharides have also been used to modify FG [14]. In particular, enzyme modification offers several advantages, including the formation of structurally stable products, superior selectivity, substrate specificity, mild reaction conditions, and high enzyme availability [15]. Transglutaminase (TG), a naturally occurring enzyme found in almost all organisms, is often used to catalyze the intermolecular and intramolecular covalent cross-linking of proteins for functional modification [16]. TG can induce the formation of ε-(γ-glutamyl)–lysine (G-L) cross-links in proteins by promoting the formation of peptide bonds between the ε-amino groups of lysine residues and γ-amide groups of glutamine residues through a three-step reaction: acyl transfer, cross-linking, and deamidation [17]. The cross-linking of TG can modify the rheological properties, gel strength, and thermo-reversibility of gelatin gels. Xu et al. [18] reported that TG cross-linking creates a denser protein network structure in porcine gelatin, successfully enhancing the thermal stability of resulting emulsions. Meanwhile, Lin et al. [19] found that TG can induce dose-dependent cross-linking, where increasing TG concentrations result in sturdier gelatin gels. Phenolic compounds are secondary plant metabolites with diverse biological functions. They exhibit antioxidant, antimicrobial, and anticancer properties, which are beneficial for human health [20]. Epigallocatechin gallate (EGCG), a polyphenol monomer found in green tea, is the most abundant bioactive tea polyphenol [21,22]. EGCG contains numerous hydroxyl groups and thus exhibits strong reactivity with other biological macromolecules, forming interactions and thereby altering their properties [23]. Consequently, EGCG is often utilized to enhance the functional properties of proteins. For instance, EGCG has been employed to improve the emulsifying properties of bovine bone protein [24], reshape the structure of soybean protein fibrils [25], and enhance the gelling properties of myofibrillar protein [26]. Both TG and EGCG are capable of inducing protein cross-linking and modifying the network structure, thereby enhancing the gel strength of gelatin. Qin et al. [27] reported that surimi gel treated with a combination of EGCG and TG exhibited greater gel strength compared to that treated with TG alone, suggesting a synergistic effect of EGCG and TG in improving gel strength. The combined application of EGCG and TG leads to a reduction in the surface hydrophobicity of myosin, total sulfhydryl content, reactive sulfhydryl content, disulfide bond content, and the proportion of α-helix structures (from 23.93% to 5.63%) [28].

The application of FG in the food industry is significantly hindered by its inferior gelation and rheological properties [29]. In this study, *Takifugu rubripes* skin was selected as a raw material for FG extraction, and *Takifugu rubripes* FG was modified using varying concentrations of TG and EGCG. This study aimed to examine the effects of TG and EGCG treatment on the conformational properties, rheological characteristics, and gel microstructure of FG. Additionally, it sought to determine the optimal conditions for the TG- and EGCG-induced modification of FG and elucidate the relationship between the treatment concentrations of TG and EGCG and the gel properties of FG.

## 2. Results and Discussion

### 2.1. Yield and Composition of FG

The FG extraction yield in this study was found to be 20.5 ± 2.49% (grams of dry gelatin per 100 g of clean skin). This was similar to the yield of tilapia FG in the study by Niu et al. [30]. The chemical composition of FG was as follows: 93.8 ± 0.31% (*w*/*w*) protein, 2.8 ± 0.05% (*w*/*w*) ash, and 1.0 ± 0.019% (*w*/*w*) lipid. These proportions indicated that water and fat were efficiently removed from the processed skin. The amino acid composition of the extracted gelatin is shown in Table 1. Glycine was the most abundant amino acid in all gelatin samples. The content of proline was 8.20 ± 0.35%, lower than that observed in porcine gelatin [31]. This was expected, as FG typically has different amino acid ratios compared to mammalian gelatin and consequently exhibits different functional properties [32].

### 2.2. Zeta Potential of FG

**Figure 1:**
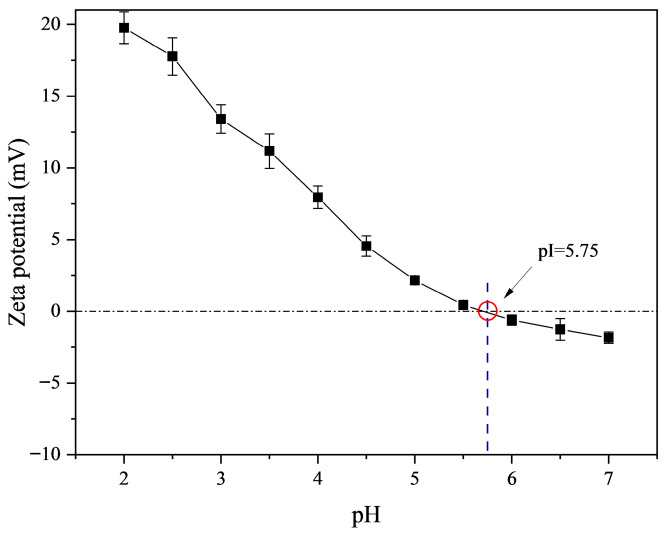
ζ-potential diagram of fish skin gelatin as a function of pH.

The zeta potential of fish skin gelatin was measured using potentiometric analysis, as illustrated in Figure 1. The pH exhibited a significant influence on the zeta potential of fish skin gelatin. As the pH decreased gradually, the zeta potential also de-creased correspondingly. When the pH reached 5.75, the zeta potential approached zero. Consequently, the isoelectric point (pI) of fish skin gelatin was determined to be approximately 5.75. Type A gelatin typically exhibits an isoelectric point ranging from 6 to 8, whereas type B gelatin has an isoelectric point within the range of 4.7 to 5.3. Based on the zeta potential analysis, the extracted redfin oriental puffer fish skin gelatin demonstrated characteristics more closely resembling those of type B gelatin.

### 2.3. Structural Properties of TG- and EGCG-Modified Gelatins

#### 2.3.1. Molecular Weight Distribution

The molecular weight distribution of TG- and EGCG-modified gelatin was examined using GPC-MALLS. Table 2 shows the molecular weight parameters of all gelatin samples. The increase in TG and EGCG treatment concentrations significantly affected the molecular weight of gelatin. The weight-average molecular weight (M_w_) of FG samples modified with TG increased significantly from 3.337 × 10^5^ Da for unmodified FG to 6.902 × 10^5^ Da for FG-TG 0.05%, and the Mean-square radius of gyration (R_g_) increased from 30.100 nm for unmodified FG to 34.600 nm for FG-TG 0.05%. Similarly, the M_w_ and R_g_ of FG samples modified with EGCG also increased to 15.756 × 10^5^ Da (in FG-EGCG 0.5%) and 35.800 nm (in FG-EGCG 0.3%), respectively. However, when the concentration of EGCG was 0.5%, R_g_ decreased to 32.268 nm. This may be because excessive amounts of EGCG induced conformational changes, leading to the formation of a compact structure in gelatin molecules. These results clearly indicate that gelatin molecules can be modified by TG and EGCG, resulting in the formation of larger molecules. Zhao et al. [33] observed that the hardness of the modified gel attained its peak value at a TG concentration of 0.01%. Guo et al. [34] investigated the effects of four different polyphenols and found that a concentration of 6 μmol/g contributed to the formation of a more homogeneous gel network.

The number-average molecular weight (M_n_) of the FG-TG samples initially decreased with increasing TG concentrations and then increased thereafter. The increase in M_w_/M_n_ indicated that TG promoted the cross-linking of gelatin molecules, enabling the integration of low-molecular-weight gelatin fragments into larger molecular structures. This further confirmed that TG first hydrolyzes protein molecules and then catalyzes cross-linking between and within protein molecules [35]. In contrast, the M_n_ of FG-EGCG samples was lower than that of unmodified FG, while the M_w_/M_n_ was higher. This indicated that EGCG likely disrupted some of the existing protein structures in gelatin or prevented protein aggregation, resulting in more low-molecular-weight fragments. Simultaneously, EGCG could interact with specific gelatin molecules through non-covalent interactions, such as hydrophobic and hydrogen bonds [36]. This created a more heterogeneous mixture of varying molecular sizes, explaining the increase in the M_w_/M_n_ ratio.

**Figure 2:**
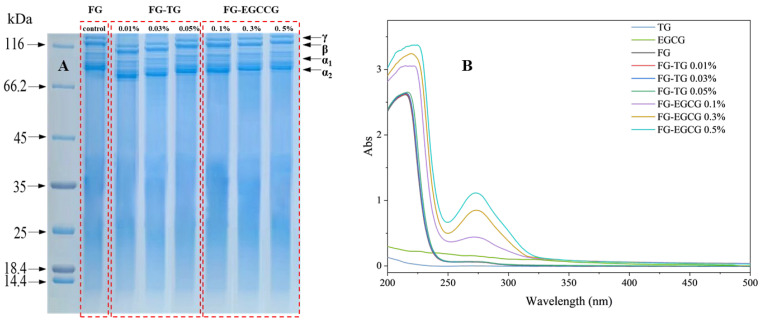
Characterization of unmodified as well as TG- and EGCG-modified gelatin. (**A**): SDS-PAGE analysis of FG following TG and EGCG addition. (**B**): Effect of TG and EGCG addition on the UV absorption spectrum of FG.

SDS-PAGE was used to determine the protein profile of TG- and EGCG-modified FG. As shown in Figure 2A, the molecular weight of FG increased with rising TG and EGCG concentrations, consistent with the GPC-MALLS results. The bands detected on SDS-PAGE indicated that *Takifugu rubripes* FG is mainly composed of γ, β, α_1_, and α_2_ subunits and contains typical collagen fragments. Notably, TG-modified FG displayed numerous protein degradation bands with a broad molecular weight distribution, reflecting a small amount of degradation and hydrolysis, probably due to high-temperature treatment [37]. This may be because high-molecular-weight polymers were generated when FG was cross-linked using TG, and these polymers accumulated around the loading wells [19]. With the increase in TG concentration, the protein content of these fuzzy bands decreased significantly, while the abundance of high-molecular-weight proteins increased, suggesting enhanced cross-linking efficiency [38]. As the TG concentration increased, the density of the γ and β chain bands began to decrease, and the additional 50–100 kDa bands became more diffuse. The γ, β, α_1_, and α_2_ chains of gelatin also showed significant degradation. During the conversion of collagen to gelatin, intramolecular and intermolecular cross-linking bonds are destroyed along with some collagen amide bonds, leading to the formation of low-molecular-weight fragments. In this study, the γ chain band of EGCG-modified FG was wider than that of unmodified FG, indicating a higher concentration of γ chains in the EGCG-treated samples. With the increase in EGCG concentration, the peak molecular weight increased, in line with the GPC-MALLS results. The increase in gel strength could be due to the formation of secondary bonds between protein molecules, including hydrogen bonds, hydrophobic interactions, and electrostatic interactions. These results collectively indicated that the polyphenols and gelatin could undergo associations [39].

#### 2.3.2. UV Spectral Analysis

The UV spectrum can determine the side chain changes in aromatic amino acids [40]. All gelatin samples exhibited significant ultraviolet absorption capacity in the range of 200–250 nm, mainly due to the characteristic absorption properties of the gelatin poly-peptide skeleton [41]. Figure 2B shows the UV absorption spectra of gelatin samples modified with different concentrations of TG and EGCG. Notably, the UV absorption spectrum of FG modified with TG was largely consistent with that of unmodified FG. Under the influence of TG, the ε-amino group of TG lysine residues may form cross-linked peptide bonds with glutamine residues, leaving other amino acids unaffected. Since only aromatic amino acids exhibit absorption capacity in the UV region and TG-catalyzed cross-linking does not affect these residues, no significant changes were observed in the UV spectrum of TG-modified gelatin. In gelatin, a distinct UV absorption peak is observed within the wavelength range of 250–290 nm, which is primarily attributed to the conjugated double bonds present in the aromatic amino acid residues of tryptophan and tyrosine [42]. As the concentration of EGCG increases, a slight redshift in the UV absorption peak of FG is detected, ranging from 271 to 273 nm. This phenomenon indicates that EGCG induces conformational changes in gelatin, thereby altering the microenvironment of its amino acid residues. Consequently, aromatic residues that were previously buried within the protein structure become exposed to a more hydrophobic environment [43]. Hydrogen bonding represents one of the primary molecular interactions between phenolic compounds and proteins and plays a crucial role in promoting protein aggregation [44], which ultimately leads to a more compact and dense microstructure in gelatin.

#### 2.3.3. Microstructural Analysis

**Figure 3:**
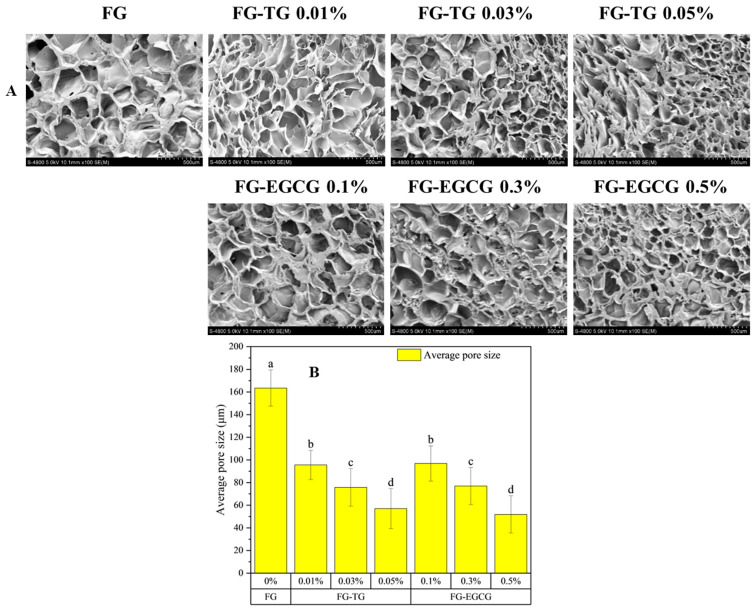
(**A**) Microstructure and (**B**) Average pore size (μm) of FG modified with different concentrations of TG and EGCG. Different letters indicate significant difference (*p* < 0.05).

SEM showed that gelatin contains an obvious three-dimensional network structure (Figure 3A). Owing to the significant amount of empty space within this structure, gelatin could easily retain water.

On a microstructural level, SEM showed that unmodified FG contains an uneven arrangement of differently sized irregular holes, possibly due to its loose internal network and insufficient strength. This also explained the low elasticity and hardness of unmodified FG. However, the network structure of FG was altered after TG and EGCG modification. As the concentration of TG and EGCG increased, the cross-sectional pores gradually became smaller, and their arrangement became denser and more uniform. The pore size of FG samples modified with TG decreased significantly from 163 μm in unmodified FG to 57 μm, while the pore size of EGCG-modified FG samples decreased to 51 μm (Figure 3B). A more compact network structure leads to more stable intermolecular binding. Previous studies on gelatin and myofibrillar proteins have demonstrated that the addition of TG and EGCG can increase the density and uniformity of the gel network [32]. However, other studies have also shown that excessive EGCG can destroy protein microstructure [34]. Collectively, on a microstructural level, the enhancement of gel properties observed after TG and EGCG treatment was consistent with the altered texture and rheological properties of FG observed in this study.

### 2.4. Physicochemical Properties of TG- and EGCG-Modified Gelatin

**Figure 4:**
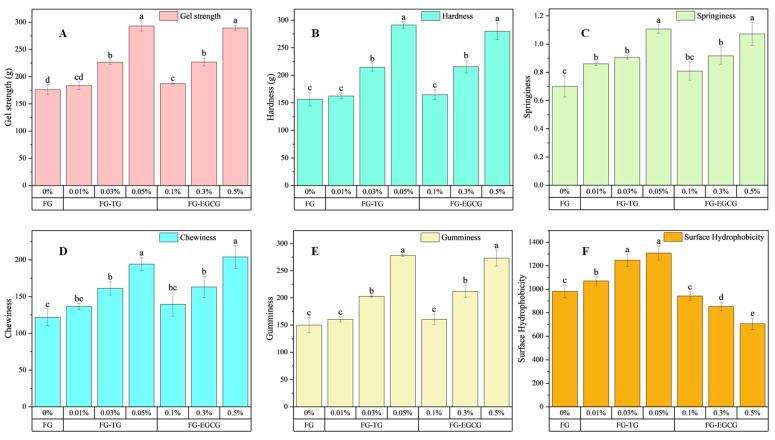
Physicochemical properties of unmodified and TG- and EGCG-modified gelatin. (**A**–**E**): Textural properties of 10% FG modified with different concentrations of TG and EGCG. (**F**): Effect of TG and EGCG modification on the surface hydrophobicity of FG. Different letters in the bar chart indicate significant differences (*p* < 0.05).

Texture is an important physicochemical property for proteins. The TPA results of FG modified using different concentrations of TG and EGCG at 25 °C are shown in Figure 4A–E. The FG modified with TG and EGCG exhibited a pale-yellow hue, characterized by excellent transparency and uniformity, with no discernible turbidity or precipitation observed. In this study, with the increase in TG and EGCG concentration, the gel strength, hardness, springiness, chewiness, and gumminess of FG increased gradually, showing significant alterations when compared to unmodified one. A previous study found that the gel strength of walleye pollock FG first increases and then decreases with the addition of EGCG [45]. This could be attributed to the adequate coating formed by polyphenol molecules on the surface of the protein. In our study, the overall concentration of EGCG may have been insufficient to induce this phenomenon. Haug et al. [46] suggested that a higher hydrophobic amino acid content can lead to higher gelatin gel strength. Therefore, the enhancement of the structural properties of gelatin could be related to the increase in hydrophobic amino acids. In addition, gel texture is also influenced by the gel network structure itself. Gels with well-organized 3D network structures are generally harder, while those with coarser and irregular structures (specifically those containing aggregation-type networks) are softer [47]. Furthermore, in this study, the triple-helix structures present in FG reduced as the amount of cross-linker increased. This clearly demonstrated that the presence of cross-links along the gelatin chain affected their ability to assemble into triple helices. These disturbances prevented orderly rearrangement, interfering with the formation of the 3D network structure of gelatin gels [48]. However, more protein aggregation with increasing concentrations of TG prevented gelatin from forming well-defined 3D structures, causing a decrease in gel hardness. Gómez-Guillén et al. [49] and Liu et al. [50] also reported that when the TG concentration exceeds the amount required for optimal cross-linking effects, the protein network structure can become excessively tight, leading to a significant decrease in gel hardness. Low gel strength can negatively impact the applications of gelatin. Therefore, TG can be used to catalyze cross-linking to improve the properties of FG, particularly its gel strength. Our findings show that the TG concentration required for cross-linking gelatin is much lower than the required EGCG concentration, indicating that TG as an enzyme provides significant advantages as a cross-linking agent. Our findings are consistent with earlier reports. Xu et al. [51] observed that TG treatment significantly increased the gel strength of mammalian and aquatic gelatins, supporting our observation that TG requires much lower concentrations than EGCG to achieve comparable improvements. In contrast, Yan et al. [45] and Li et al. [36] demonstrated that EGCG enhances the gelation properties of gelatin and myofibrillar proteins in a concentration-dependent manner, which explains the moderate improvements observed in our study and the potential destabilization at higher EGCG levels. These comparisons indicate that TG acts as a more efficient cross-linking agent, whereas EGCG provides supplementary effects through polyphenol–protein interactions. Together, these findings highlight that TG and EGCG can be applied as natural and safe modifiers in food gelatin systems, but their efficiency and mechanisms differ significantly.

Surface hydrophobicity (H_0_) reflects the number of hydrophobic groups exposed on a protein’s surface and acts as a crucial determinant of its emulsifying properties. In the present study, the H_0_ of TG-FG was found to be higher than that of FG. Moreover, the H_0_ of TG-modified FG increased proportionally with the rise in TG concentration (Figure 4F). This increase could be attributed to two factors: first, the slight degradation of high-molecular-weight peptides, exposing more hydrophobic amino acids [52], and second, the significant molecular aggregation behavior of gelatins after TG modification. Xu et al. [51] demonstrated that TG treatment can increase the H_0_ of both mammalian and aquatic gelatins. In the TG-catalyzed reaction process, ε-(γ-glutamyl)-lysine isopeptide bonds can be generated and inserted into the triple-helix structure, resulting in a more open protein configuration [53]. This leads to the exposure of hydrophobic residues that were previously buried within the interior of gelatin molecules. Additionally, the loss of ε-amino groups from lysine residues during TG-catalyzed reactions reduces the hydrophilicity of these amino acids [38]. However, Lin et al. [19] also showed that TG can reduce the H_0_ of pure tilapia FG due to the exposure of more hydrophilic amino acids [54]. These differences may arise from the varying ratios of hydrophilic and hydrophobic amino acids in different types of FG.

Notably, EGCG modification led to significantly lower H_0_ values in FG than TG modification. Research has shown that EGCG can alter the spatial configuration of proteins, causing more amino acid residues to be exposed and increasing potential interactions [55]. With the increase in EGCG concentration, the H_0_ of FG appeared to decrease, indicating that EGCG may bind to the exposed amino acid residues via different molecular forces. Phenolic compounds are known to expose hydrophobic amino acid residues in proteins [56], thereby enhancing the interaction between them. This further alters the structure of proteins and improves their performance. Similar results were also reported in previous studies on myofibrillar protein and whey protein isolate, where the covalent interactions between ECCG and myofibrillar protein [57] or whey protein isolate [58] reduced the surface hydrophobicity of the protein, in line with the results of our study.

### 2.5. Rheological Property Determination

#### 2.5.1. Frequency Sweep Tests

**Figure 5:**
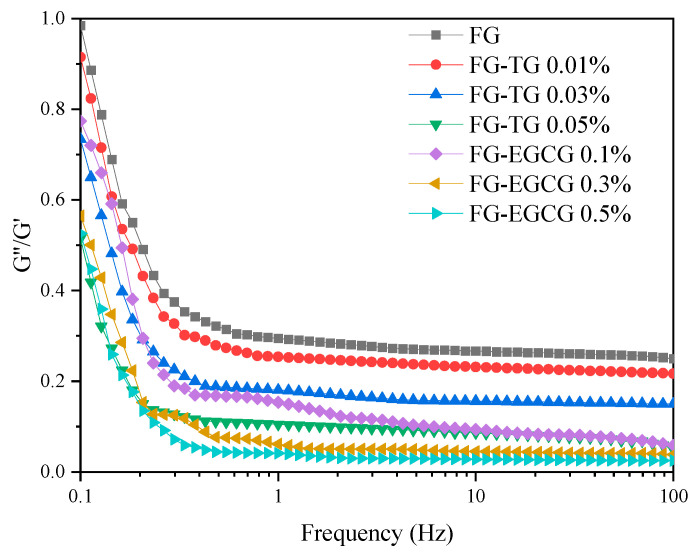
Effect of TG and EGCG addition on the tanδ of FG.

Frequency sweeps were performed over a range of 0.1–100 Hz under 0.5% strain to determine the dependence of G″/G′ (tanδ) on frequency (Figure 5). All the mixtures exhibited higher G′ than G″ values in the frequency range of 0.1–100 rad/s, resulting in tanδ values less than 1. This was indicative of elastic dominance in the system and confirmed that all the mixtures existed as interconnected network structures with good stability against mechanical stress [59]. After TG and EGCG were added, they interacted with gelatin molecules through intermolecular forces to form a three-dimensional network structure that was denser and more ordered. With the increase in TG and EGCG concentrations, tanδ values decreased gradually. At the same oscillation frequency, the addition of 0.05% TG and 0.5% EGCG yielded lower tanδ values. Therefore, we concluded that higher TG and EGCG concentrations led to the faster interactions of molecules within gelatin and a more rapid increase in G′. In addition, the gel strength was higher when the concentration of TG and EGCG was greater.

#### 2.5.2. Temperature Sweep Tests

**Figure 6:**
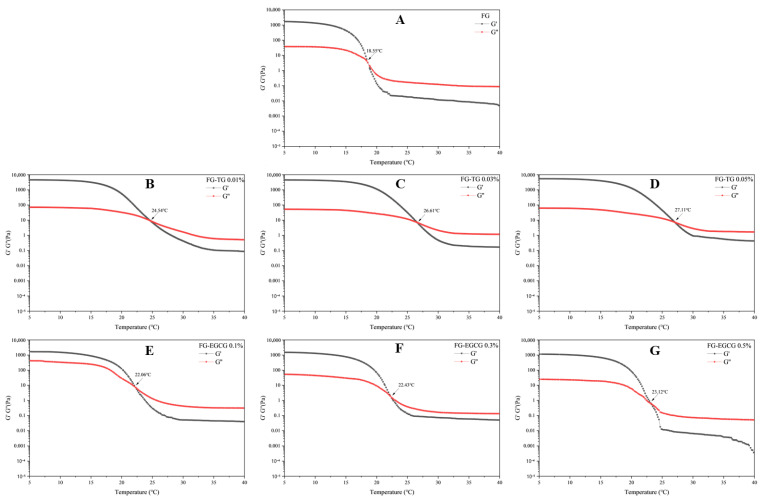
Temperature sweep tests of FG modified with TG and EGCG (f = 1 Hz; γ = 0.5%). (**A**–**G**) represent the melting temperatures (T_melt_) of FG modified with varying TG and EGCG concentrations.

The thermodynamic and mechanical stability of gelatin gels was studied by performing temperature sweep tests from 5 °C to 40 °C. The melting point is a critical parameter that reflects the thermal stability of a gel and is commonly defined as the point where G′ and G″ intersect [38]. As shown in Figure 6, the melting point of FG was 18.55 °C. Liao et al. [60] reported a melting temperature range of 24.76–31.29 °C for tilapia FG, depending on its extraction temperature and pH. Typically, FG shows a lower melting point than porcine (31 °C) and bovine skin gelatin (29 °C) due to its lower content of proline, hydroxyproline, and hydrophobic amino acids [38]. With the increase in TG and EGCG concentration, however, the melting point of FG increased gradually. The highest melting points detected after TG and EGCG modification were 27.11 °C and 23.12 °C, respectively. The elevation in the melting point of FG allows the modified FG to be stably stored at room temperature (25 °C), facilitating its processing into meat jelly-like products suitable for commercial distribution and consumption. These results suggested that TG and EGCG addition can improve the thermodynamic and mechanical stability of FG gels by introducing cross-links that contribute to the formation of more ordered structures [19,61].

#### 2.5.3. Steady Shear Flow Tests

**Figure 7:**
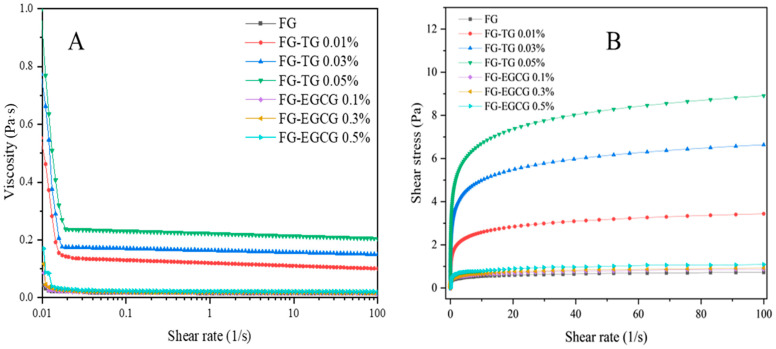
Effect of TG and EGCG modification on the viscosity and shear stress of FG.

The influence of TG and EGCG addition on the steady shear properties of FG is shown in Figure 7. The addition of different concentrations of TG and EGCG significantly increased the viscosity of FG (Figure 7A). The viscosity of all samples decreased with increasing shear rates, exhibiting shear-thinning behavior. This phenomenon likely occurred because at high shear rates, a greater amount of shear force was applied to the gel system, limiting the organization of large networks in FG [62].

Notably, the shear stress gradually increased with increasing TG concentrations at all shear rates, indicating an increase in dispersion viscosity (Figure 7B). The quality of FG improved as the TG concentration increased, demonstrating that TG can significantly enhance the properties of gelatin. Although the properties of FG also improved following EGCG modification, samples treated with 0.05% TG showed better properties than those treated with 0.5% EGCG. This indicated that TG was more efficient at modifying the properties of FG.

## 3. Conclusions

This study examined the effect of TG and EGCG modification on the rheological, structural, and physicochemical properties of *Takifugu rubripes* FG. With increasing TG and EGCG concentrations, more interactions occurred in FG, and its properties were significantly improved. After modification, the molecular weight of FG proteins increased, and the surface hydrophobicity and gel strength also showed improvements. Rheological analysis showed that the viscosity of TG- and EGCG-modified FG was also higher than that of unmodified FG and was positively correlated with the concentration of TG and EGCG. Notably, TG demonstrated higher efficiency as a cross-linking agent when compared to EGCG, requiring lower concentrations to achieve similar improvements in gelatin properties. However, this study has certain limitations, including the focus on a single fish species and the evaluation of modifications under laboratory conditions only. Future research should investigate the stability and performance of modified FG under various storage conditions, explore the effects of combination treatments with other natural cross-linking agents, and conduct sensory evaluation and shelf-life studies to assess the practical applicability of these modifications in commercial food products. The findings of this study provide valuable insights that could guide the development of FG-based products with enhanced functional properties and aid their applications in food packaging, edible films, and emulsion-based delivery systems.

## 4. Materials and Methods

### 4.1. Materials

*Takifugu rubripes* skin was supplied by Dalian Tianzheng Industry Co., Ltd. (Dalian, China). TG was obtained from Sunson Industry Group Co., Ltd. (Cangzhou, China), while EGCG was acquired from Shanghai Macklin Biochemical Technology Co., Ltd. (Shanghai, China). All other chemical reagents were procured from Tianjin Kemiou Chemical Reagent Co., Ltd. (Tianjin, China).

### 4.2. Extraction of Takifugu rubripes FG

Frozen pieces of *Takifugu rubripes* skin were thawed at 4 °C for 20 h, de-scaled, and cut into small squares (approximately 2 × 2 cm^2^) immediately before gelatin extraction. The square pieces were soaked in 0.1 mol/L NaOH solution with stirring for 8 h, where the solution was replaced every 2 h (skin-to-NaOH ratio of 1:10 *w*/*v*). Subsequently, the skin pieces were thoroughly washed with water until a neutral pH of 7 was achieved. The samples were then soaked in 0.2% H_2_SO_4_ solution for 2 h (1:6 *w*/*v*) before washing with water until a neutral pH was achieved. Next, the samples were treated with 1.0% citric acid solution for 2 h (1:6 *w*/*v*) and subsequently rinsed with water until a neutral pH was attained. Following acid treatment, the samples were subjected to degreasing in a 10% n-butanol solution (1:10 *w*/*v*) for 24 h, during which the solution was changed every 8 h. Finally, FG was extracted using water at 60 °C for 6 h (skin-to-water ratio of 1:5 *w*/*v*). The resulting mixture was centrifuged at 8000 rpm, and the supernatant was freeze-dried (FD-1C-50+, BoYiKang Instrument Co., Ltd., Beijing, China) to yield the gelatin powder.

### 4.3. Analysis of the Basic Components of Gelatin

First, 0.5 g of the extracted FG powder was hydrolyzed for 22 h using 6 mol/L HCl in an oil bath at 110 °C. After cooling to 25 °C, the mixture was centrifuged, and the supernatant was collected and dried under a stream of nitrogen gas. The dried sample was subsequently redissolved in 1 mL of 0.02 mol/L HCl. The resulting solution was filtered through a 0.22-µm membrane, and the amino acid composition of the filtrate was analyzed using an automated amino acid analyzer [31] (SKD-1000; Shanghai Peiou Analytical Instruments Co., Ltd., Shanghai, China).

The fat, protein, and ash contents of FG were determined according to AOAC 2000 [63]. The crude protein content was measured using the micro-Kjeldahl method. The ash content was determined by incinerating the sample in a muffle furnace (SX-4-10; Shanghai Techeng Machinery Equipment Co., Ltd., Shanghai, China) at 550 ± 50 °C until a white ash residue was obtained. Meanwhile, the total lipid content was determined using the Soxhlet extraction method using diethyl ether as the solvent.

### 4.4. Zeta Potential Measurements

The zeta potential of fish skin gelatin was measured using a potentiometric analyzer, according to the method described by Wu et al. [64]. Briefly, the initial pH of a 10 g sample (0.1%, *w*/*w*) was adjusted to 7.0 with 1.0 M NaOH. Subsequently, titration was performed using 0.25 M NaOH and HCl solutions of 0.25 M and 0.5 M concentrations, respectively, across a pH range of 7.0 to 2.0 with an interval of 0.5. The change in zeta potential as a function of pH was recorded. All measurements were conducted at 25 ± 0.1 °C, and each sample was analyzed in triplicate, with the average value re-ported.

### 4.5. Preparation of TG- and EGCG-Modified FG Samples

*Takifugu rubripes* FG (10%, *w*/*w*) was prepared by dissolving gelatin powder in deionized water that had been boiled and subsequently cooled to room temperature. The pH of the resulting concentrated gelatin solution was adjusted to a range of 5–6. Subsequently, the solution was sealed in 20 mL glass vials and magnetically stirred at 40 °C for 30 min. Appropriate amounts of TG (1000 U/g) and EGCG were added to achieve TG concentrations of 0.01% (*w*/*w*), 0.03% (*w*/*w*), and 0.05% (*w*/*w*) as well as EGCG concentrations of 0.1% (*w*/*w*), 0.3% (*w*/*w*), and 0.5% (*w*/*w*). The mixtures were placed in a 40 °C water bath for 30 min to ensure complete dissolution. Subsequently, the solutions were transferred to a 70 °C water bath for 5 min to inactivate the enzymes. Finally, the prepared solutions were freeze-dried to obtain gelatin samples modified via treatment with varying concentrations of TG and EGCG.

### 4.6. Gel Permeation Chromatography–Multi-Angle Light Scattering (GPC-MALLS) Analysis

The molecular weights of TG- and EGCG-modified FG samples were determined using GPC-MALLS (DAWN HELEOS, Wyatt Technology, Santa Barbara, CA, USA) according to the method reported by Hu and colleagues [65]. Briefly, a NaCl solution (0.2 mol/L) filtered through a 0.22-μm Millipore filter was used as the eluent at a constant flow rate of 0.4 mL/min at 25 ± 2 °C. The sample solutions were injected into the GPC-MALLS system after proper dilution and filtration through a 0.45-μm Nylon filter. Data were collected and analyzed using Astra 5.3.4.14 software.

### 4.7. Physicochemical and Structural Properties of TG- and EGCG-Modified Gelatin

#### 4.7.1. Sodium Dodecyl-Sulfate Polyacrylamide Gel Electrophoresis (SDS-PAGE)

The molecular weight distribution of the TG- and EGCG-modified FG samples was determined using Tricine-SDS-PAGE according to the method reported by Schägger and Jagow [66]. Briefly, the samples were dissolved in a solution containing 2% (*w*/*w*) SDS, 0.5 mol/L Tris-HCl (pH 6.8), 25% (*w*/*w*) glycerol, and 5% (*w*/*w*) β-mercaptoethanol. The samples were electrophoresed on a 6% (*w*/*v*) separating gel and a 5% (*w*/*v*) stacking gel at a constant voltage of 120 V (DYY-6D, Beijing Liuyi Biotechnology Co., Ltd., Beijing, China). After electrophoresis, the gel was stained with 0.05% Coomassie brilliant blue R-250 (Shanghai Aladdin Bio-Chem Technology Co., Ltd., Shanghai, China) and then destained with 50% ethanol and 9% acetic acid. When clear bands were observed, the gel was removed and photographed using a digital camera.

#### 4.7.2. Surface Hydrophobicity (H_0_)

The H_0_ values of the TG- and EGCG-modified FG samples were measured using 8-anilino-1-naphthalenesulfonic acid (ANS) as a fluorescent probe. As described by Luo et al. [67], with some modifications, samples were serially diluted with 20 mM phosphate buffer (pH 7) to achieve protein concentrations of 0.0125, 0.025, 0.05, 0.1, and 0.2 mg/mL. These samples were then mixed with 8 mmol/L ANS at a volume ratio of 200:1. The fluorescence intensity within the wavelength range of 470–600 nm was examined after excitation at 390 nm using an RF-5301PC fluorescence spectrophotometer (Shimadzu Corporation, Kyoto, Japan). The initial slope of fluorescence intensity versus protein concentration was calculated based on linear regression analysis and used to measure H_0_.

#### 4.7.3. Ultraviolet (UV) Spectral Analysis

The TG- and EGCG-modified gelatin samples were used to prepare 0.05% solutions, which were added to a quartz cuvette for UV analysis (TCC-240AA, Shimadzu Corporation, Kyoto, Japan). The UV absorption spectra were recorded in the wavelength range of 200–500 nm. The scanning speed was 200 nm/min and the test temperature was 25 ± 2 °C. Deionized water was used as the blank control.

#### 4.7.4. Texture Profile Analysis (TPA)

The texture properties of TG- and EGCG-modified gelatin were measured using a texture analyzer equipped with the P/5 probe and a load cell of 5 kN (TA XT Plus, Lotun Science Co., Ltd., Shanghai, China). The probe speed was maintained at 1 mm/s before, during, and after measurement; the deformation ratio was 40%; and the trigger force was 5 g. Each sample was tested five times, and the average value was taken. The tested textural parameters included gel strength, hardness, springiness, chewiness, and gumminess.

### 4.8. Rheological Properties

A RheoWin MARS 40 Rheometer (Thermo Fisher Scientific, Waltham, MA, USA) equipped with a parallel plate (35 mm in diameter, 0.25 mm parallel plate gap) was used to determine the rheological properties of FG samples. The strain amplitude was maintained at 0.5% within the linear viscoelastic range (LVR). Frequency sweep tests were conducted over a frequency (f) range of 0.01–100 Hz at a temperature (T) of 25 °C [68]. The measured parameters included storage modulus (G′), loss modulus (G″), and loss tangent (tanδ = G″/G′). Meanwhile, temperature sweep tests were also conducted to examine the melting temperature (T_melt_) of the samples [69] by heating from 5 °C to 40 °C at a heating rate of 0.5 °C/min under a strain of 0.5% and frequency of 1 Hz. Finally, steady shear flow tests were conducted over a shear rate range of 0.01–100 s^−1^ to obtain flow curves at a constant temperature of 25 °C [70]. The viscosity versus shear rate data were fitted based on the power law model. During all rheological experiments, silicone oil was applied around the perimeter of the samples to prevent water loss.

### 4.9. Scanning Electron Microscopy (SEM)

The TG- and EGCG-modified gelatin samples were attached to double-sided adhesive tape and coated with gold. The samples were subsequently observed using an S-4800 scanning electron microscope (SEM, Hitachi, Tokyo, Japan) at an acceleration voltage of 5 kV [52].

### 4.10. Statistical Analysis

Three parallel experiments were carried out in most cases to allow the analysis in triplicate. In contrast, TPA measurements were repeated five times. The results are reported as the mean ± standard deviation. One-way analysis of variance (ANOVA) and Duncan’s multiple range test were performed using SPSS Statistics 19 software (IBM, Chicago, IL, USA) to determine significant differences (*p* < 0.05) within and between test groups.

## Figures and Tables

**Table 1:** Amino acid composition of *Takifugu rubripes* skin gelatin.

Amino Acid	% of Total Protein
Aspartic acid	3.77 ± 0.42
Threonine	1.63 ± 0.21
Serine	2.68 ± 0.09
Glutamic acid	6.40 ± 0.11
Glycine	15.14 ± 0.67
Alanine	6.70 ± 0.15
Valine	1.63 ± 0.08
Methionine	1.19 ± 0.06
Isoleucine	0.71 ± 0.02
Leucine	1.44 ± 0.05
Tyrosine	0.46 ± 0.03
Phenylalanine	1.34 ± 0.04
Lysine	2.48 ± 0.08
Histidine	0.60 ± 0.01
Arginine	5.40 ± 0.26
Proline	8.20 ± 0.35
Total of 16 amino acids	59.77 ± 0.90

**Table 2:** Molecular weight characterization of FG modified with TG and EGCG.

TG/EGCG Concentration (%)	M_n_ (×10^5^ Da)	M_w_ (×10^5^ Da)	Polydispersity Index (M_w_/M_n_)	R_g_ (nm)
FG 0.00	1.410 ± 0.050 ^c^	3.337 ± 0.026 ^f^	2.368 ± 0.056 ^f^	30.100 ± 0.003 ^d^
FG-TG 0.01	1.293 ± 0.029 ^d^	3.798 ± 0.019 ^e^	2.939 ± 0.035 ^e^	32.600 ± 0.002 ^c^
FG-TG 0.03	1.780 ± 0.032 ^b^	5.567 ± 0.021 ^d^	3.127 ± 0.038 ^d^	32.500 ± 0.002 ^c^
FG-TG 0.05	1.861 ± 0.027 ^a^	6.902 ± 0.015 ^c^	3.710 ± 0.030 ^c^	34.600 ± 0.001 ^b^
FG-EGCG 0.1	1.190 ± 0.031 ^e^	3.856 ± 0.029 ^e^	3.240 ± 0.040 ^d^	32.800 ± 0.072 ^c^
FG-EGCG 0.3	0.989 ± 0.037 ^g^	7.834 ± 0.028 ^b^	7.921 ± 0.051 ^b^	35.800 ± 0.093 ^a^
FG-EGCG 0.5	1.038 ± 0.055 ^f^	15.756 ± 0.031 ^a^	15.181 ± 0.104 ^a^	32.268 ± 0.105 ^c^

Different letters in the same column indicate significant differences (*p* < 0.05); Data are given as mean values ± standard deviation.

## Data Availability

The data presented in this study are available on request from the corresponding authors.
